# Risk and benefit for umbrella trials in oncology: a systematic review and meta-analysis

**DOI:** 10.1186/s12916-022-02420-2

**Published:** 2022-07-08

**Authors:** Karolina Strzebonska, Mateusz Blukacz, Mateusz T. Wasylewski, Maciej Polak, Bishal Gyawali, Marcin Waligora

**Affiliations:** 1 Research Ethics in Medicine Study Group (REMEDY), Faculty of Health Sciences, Jagiellonian University Medical College, Kraków, Poland; 2 Institute of Psychology, University of Silesia, Katowice, Poland; 3 Institute of Public Health, Faculty of Health Sciences, Jagiellonian University Medical College, Kraków, Poland; 4 Department of Epidemiology and Population Studies, Institute of Public Health, Faculty of Health Sciences, Jagiellonian University Medical College, Kraków, Poland; 5 Department of Oncology and the Department of Public Health Sciences, Queen’s University, Kingston, Ontario Canada

**Keywords:** Umbrella trial, Risk-benefit balance, Ethics, Targeted therapy

## Abstract

**Background:**

Umbrella clinical trials in precision oncology are designed to tailor therapies to the specific genetic changes within a tumor. Little is known about the risk/benefit ratio for umbrella clinical trials. The aim of our systematic review with meta-analysis was to evaluate the efficacy and safety profiles in cancer umbrella trials testing targeted drugs or a combination of targeted therapy with chemotherapy.

**Methods:**

Our study was prospectively registered in PROSPERO (CRD42020171494). We searched Embase and PubMed for cancer umbrella trials testing targeted agents or a combination of targeted therapies with chemotherapy. We included solid tumor studies published between 1 January 2006 and 7 October 2019. We measured the risk using drug-related grade 3 or higher adverse events (AEs), and the benefit by objective response rate (ORR), progression-free survival (PFS), and overall survival (OS). When possible, data were meta-analyzed.

**Results:**

Of the 6207 records identified, we included 31 sub-trials or arms of nine umbrella trials (*N* = 1637). The pooled overall ORR was 17.7% (95% confidence interval [CI] 9.5–25.9). The ORR for targeted therapies in the experimental arms was significantly lower than the ORR for a combination of targeted therapy drugs with chemotherapy: 13.3% vs 39.0%; *p* = 0.005. The median PFS was 2.4 months (95% CI 1.9–2.9), and the median OS was 7.1 months (95% CI 6.1–8.4). The overall drug-related death rate (drug-related grade 5 AEs rate) was 0.8% (95% CI 0.3–1.4), and the average drug-related grade 3/4 AE rate per person was 0.45 (95% CI 0.40–0.50).

**Conclusions:**

Our findings suggest that, on average, one in five cancer patients in umbrella trials published between 1 January 2006 and 7 October 2019 responded to a given therapy, while one in 125 died due to drug toxicity. Our findings do not support the expectation of increased patient benefit in cancer umbrella trials. Further studies should investigate whether umbrella trial design and the precision oncology approach improve patient outcomes.

**Supplementary Information:**

The online version contains supplementary material available at 10.1186/s12916-022-02420-2.

## Background

Precision oncology is a strategy aiming to divide cancer patients into groups that will most likely respond to a given therapy. Treatment is tailored to the molecular makeup of a tumor rather than the site or stage of disease [1].

Umbrella trials are novel trial designs commonly used in precision oncology [2, 3] defined as trials with “many different treatment arms within one trial. People are assigned to a particular treatment arm of the trial based on their type of cancer and the specific molecular makeup of their cancer” [4].

The umbrella design is a type of master protocol which allows for testing multiple agents simultaneously and may include specified modifications while the trial is ongoing [5–10]. For example, adaptive randomization is often used to assign patients to the most effective experimental treatment based on continuous data accumulation and interim analyses [11]. These features of umbrella design are considered as having the potential to accelerate the process of drug development and maximize the benefits for trial participants [12]. Umbrella trials may be also classified as platform trials [7, 13] when participants are adaptively randomized and the protocol permits considerable flexibility to add new arms when novel targets and drugs are identified or to discontinue arms with ineffective treatments [5, 8, 10, 14]. However, some researchers argue that the prospect of patient clinical benefit from umbrella trials is limited [15–18]. Since umbrella trials’ implementation in 2006 [19], many statistical objections [20] and ethical challenges have been identified [8, 21].

A favorable risk-benefit ratio is one of the fundamental ethical requirements of conducting research with human participants [22, 23]. The evaluation of risks and potential benefits to study participants requires a careful ethical analysis based on relevant data [24, 25]. The safety and toxicity rates of anticancer agents in standard phase I–III clinical trials have already been estimated [26–28]. The recent analyses were focused on targeted therapies [29, 30] which play an important role in precision medicine [31]; the performance of targeted therapies is enhanced when used in combination with cytotoxic drugs [32]. However, the only RCT in precision oncology was negative for survival [33]. Yet, little is known about the risk-benefit profile for umbrella oncology trials. The objective of our systematic review with meta-analysis was to evaluate the risks and benefits in umbrella clinical trials testing targeted drugs or a combination of targeted agents with chemotherapy. Specifically, our analysis addresses four issues: (1) the utility of a new strategy of clinical trials (umbrella designs) in oncology, (2) the utility of precision oncology, (3) the utility of pooling populations across arms and across chemotherapies, and (4) the likelihood that a drug works in more than one specific population.

## Methods

We followed the Preferred Reporting Items for Systematic Reviews and Meta-Analyses (PRISMA) 2020 guidelines [34, 35].

### Eligibility criteria

The inclusion and exclusion criteria were defined prospectively in the study protocol [36], and they are summarized in Table 1. The key inclusion criteria were as follows: (1) cancer umbrella clinical trials as defined by the American Society of Clinical Oncology [4], platform umbrella trials, or sub-studies being a part of the cancer umbrella trial; (2) adult or mixed population studies in which at least 50% participants were ≥ 18 years; (3) patients were diagnosed with any malignancy (solid or hematological) at any stage; and (4) assessment of drug-related toxicity and/or response of targeted therapy drug/s (monoclonal antibodies or small molecules or antibody-drug conjugates [37]) or a combination of targeted therapy with chemotherapy regimens in at least one experimental arm or sub-trial. We excluded studies evaluating the following: (1) hormone therapies, immunotherapies (e.g., monoclonal antibodies that were also immunotherapy), or chemotherapy only regimens; (2) a combination of targeted therapy with immunotherapy (response profile in such combination is much different from targeted solo therapies, making comparisons with regimens included in the review impossible); and (3) non-pharmacological modalities (e.g., radiotherapy, surgery, stem cell therapy, or any of these, except for targeted therapy, combined with surgery).

**Table 1:** Inclusion and exclusion criteria

Category	Inclusion criteria	Exclusion criteria
**P**	1. Studies in which at least 50% of participants were 18 years old or older and the study was not indicated as pediatric. 2. Patients with a single type of solid tumor or hematological malignancy at any stage	1. Pediatric studies. 2. Patients with benign tumors or other diseases only, without cancer.
**I**	1. Studies that test agents based on the molecular profiling of an individual patient’s tumor, defined as a method of testing genetic characteristics as well as any unique biomarkers of a cancerous tumor. The results are used to identify and create targeted therapies that work most effectively for specific cancer tumor profiles [4].	1. Studies that did not test agents based on the tumor molecular profiling.
	2. Multiple molecularly targeted therapies (monoclonal antibodies or small molecule or antibody-drug conjugates). 3. Combination of both: molecularly targeted therapies and chemotherapy. 4. Targeted therapy combined with surgery.	2. Chemotherapy only in the experimental arm—cytotoxic drug schedules, monotherapy, or polytherapy. 3. Studies in which hormone therapy, immunotherapy, surgery, or radiotherapy were the only treatment. 4. Radiotherapy or immunotherapy used together with targeted therapy. 5. Supportive care without anticancer agents and other types of drugs and treatments, i.e., antiviral agents or non-specific immunotherapy (e.g., interferon, interleukins, cytokines, immunostimulator, GM-CSF granulocyte-macrophage colony-stimulating factor), cancer vaccine, and oncolytic virus therapy.
	5. Drugs administered systemically.	6. No systemic administration (e.g., topical only).
**C**	1. Standard of care/placebo. 2. Experimental arm only in case of a non-match arm.	–
**O**	1. Measures of benefit: objective response rate or progression-free survival. 2. Measures of risk: grade 3, 4, or 5 drug-related events. 3. Additional outcomes: disease control rate, overall survival, time to progression, and duration of response.	1. No data on measures of benefit and/or risk.
**S**	1. Umbrella trials defined as studies that have many different treatment arms within one trial (participants are assigned to a particular treatment arm of the trial based on their type of cancer and the specific molecular makeup of their cancer) [4]. 2. Sub-studies testing targeted therapy or a combination of targeted therapy and chemotherapy that were part of the umbrella master protocol. 3. Platform umbrella trials as classified by the study authors and/or umbrella trials utilizing Bayesian response-adaptive randomization and/or umbrella trials in which sub-trials are added or suspended continuously [7, 13]. 4. Interventional studies of all phases (i.e., I, II, III). 5. Publications of sub-studies that were part of one umbrella study.	1. Studies without umbrella design (e.g., studies testing only one targeted therapy based on the patient’s molecular makeup). 2. Studies that tested targeted therapies on multiple tumor types. 3. Observational studies, review articles, and articles describing only umbrella study design without results.
	6. Full articles and abstracts.	4. No full text available.

*P* population, *I* intervention, *C* comparator, *O* outcomes, *S* study type

### Data sources and search strategy

We systematically searched Embase and PubMed for umbrella trial articles and abstracts published between 1 January 2006 and 7 October 2019, using strategies that included keywords and suggested MeSH and Emtree entry terms, their synonyms, and closely related words (Additional file 1: Table S1). Searches were not limited by language. The starting date of our search period was determined by the year of launching the first umbrella study [19]. Our search strategies were checked using the Canadian Agency for Drugs and Technologies in Health peer-review checklist for search strategies [38].

### Study selection process

Two experienced coders (KS, MTW) independently screened the records for the initial study inclusion and performed a full-text screening to determine the final inclusions. Disagreements were resolved by discussion, and when necessary, a third person, an arbiter, was involved (MW).

### Data extraction

We created and piloted a data extraction form. Based on the pilot, we refined and prepared the final version (available from the Open Science Framework (OSF), https://osf.io/kuyaz/). Data were extracted from each publication independently by two reviewers (KS, MB). Discrepancies were resolved by discussion, and when necessary, an arbiter was involved (MW). An experienced medical oncologist had a supervisory role (BG). In the case of multiple publications for the same study, the results from the full publication and/or the most recent version were used in the extraction. If the NCT number was provided, additional information was searched and extracted from ClinicalTrials.gov.

**Fig. 1:**
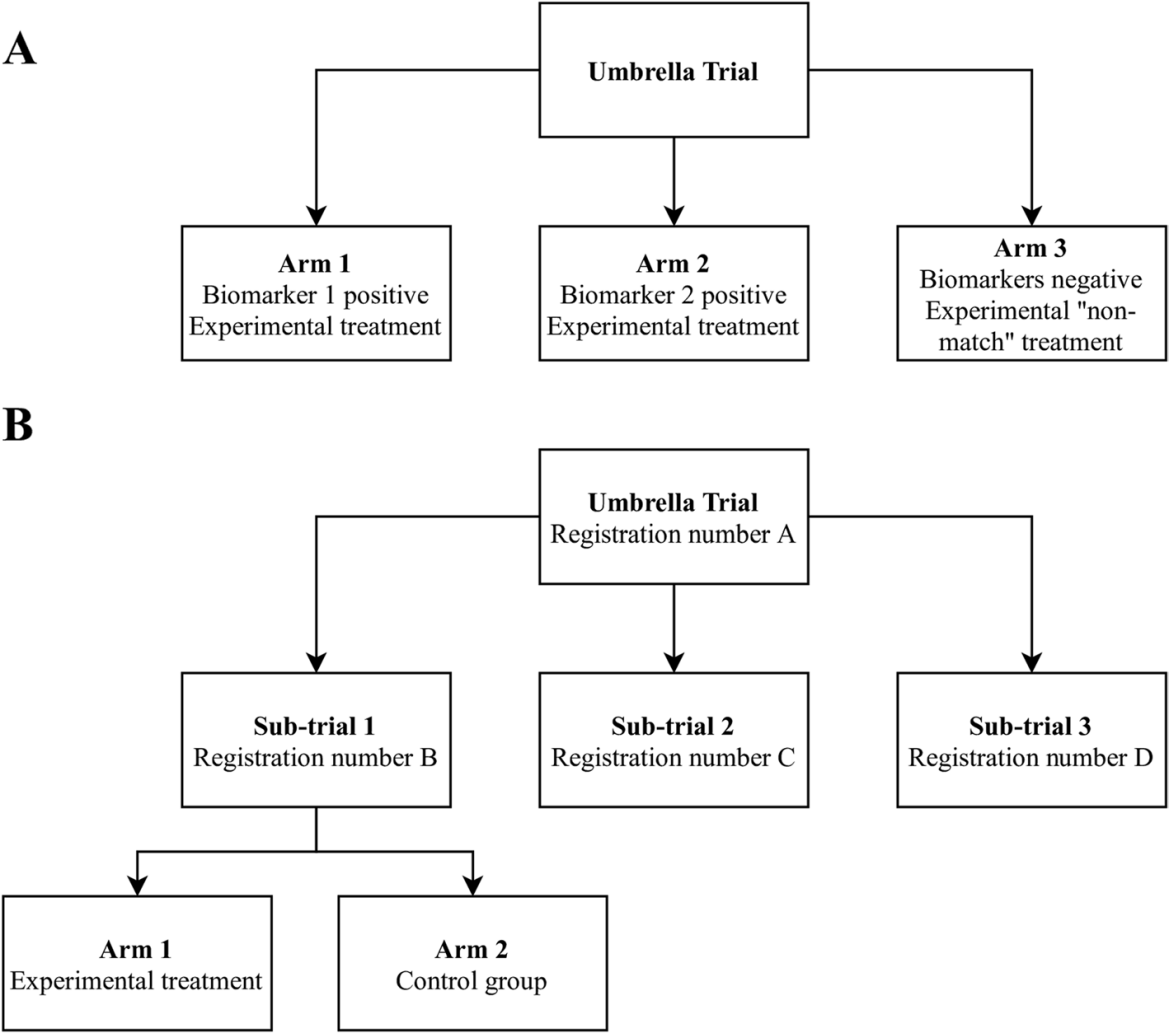
Structure types of umbrella trials. **A** An umbrella trial with multiple arms. Accrual to each arm is based on the biomarker test result. **B** An umbrella trial with multiple sub-studies. Each sub-study has a separate registration number and may include an experimental arm and a control group

Umbrella trials are very heterogeneous; some of them are studies with multiple arms (Fig. 1A), and others have a hierarchical structure with sub-trials having a unique registration number (Fig. 1B). We extracted data only from the arms or sub-trials testing targeted therapy drugs or a combination of targeted therapy with chemotherapy. If the umbrella trial or sub-trial included a placebo, control group, or non-match arm, data from these arms were extracted separately for further comparison of matched versus non-matched therapy.

We considered the therapy as “matched” to the disease when at least one tested agent was administered based on the specific molecular features of the patient’s tumor, e.g., a drug matched to the specific genetic change. If patients were treated in (1) biomarker-negative sub-study/arm, (2) so-called non-match sub-study/arm (defined as the arm or sub-study recruiting patients that did not match any of the prespecified biomarkers), (3) placebo, or (4) control group testing only chemotherapy agents, we considered these therapies as not matching the specific tumor molecular characteristics.

For each arm or sub-study, we extracted data related to study characteristics (e.g., phase, location, study status), patient characteristics (e.g., number of enrolled and eligible participants, type of malignancy), intervention (e.g., therapy type, agent names), and outcomes (e.g., objective responses, drug-related adverse events). For more details, see our extraction form (https://osf.io/kuyaz/).

### Data curation

We defined a “sub-study” of the umbrella trial as a separate trial within the umbrella protocol with a unique registration number provided by the study authors. In cases where the separate registration number was not provided, we used the term “arm.” The glossary of key manuscript terms is presented in the online appendix (Additional file 1: Table S2).

Umbrella trials generally measure short-term clinical outcomes to yield information about preliminary drug efficacy [8]. We included various measures of clinical benefit reported in umbrella trials: we classified the objective response rate (ORR) and progression-free survival (PFS) as proxies of therapeutic benefit and overall survival (OS) as the direct measure of clinical benefit. We defined the objective response rate as the proportion of participants with partial and/or complete response (reported separately or as an objective response rate) as defined by the study authors. For PFS and OS analyses, we used medians provided by the study authors.

Risks were assessed in terms of patients experiencing severe adverse events, such as the proportion of participants experiencing grade 3, 4, or 5 drug-related AEs as defined by the Common Toxicity Criteria for Adverse Events, version 5.0 (and earlier versions) [39]. An AE was considered as related to the study drug if it was clearly stated by the study authors; expressions such as “AEs attributed to treatment” and “AEs possibly, probably, or definitely related to study drug” were also acceptable. In cases where an event was not clearly described as treatment-related, we excluded it from our risk analysis.

### Risk of bias assessment

Two authors (KS, MB) independently assessed the risk of bias for all included studies using the Cochrane risk of bias tools for randomized or non-randomized studies [40, 41]. Every sub-trial/arm was assessed separately by reading all relevant literature. Judgments were based on the algorithms proposed by the authors of ROBINS and RoB2 tools, adjusted to fit the specific aspects of our analysis. Disagreements were resolved by discussion.

### Statistical analysis

Objective response rates, treatment-related fatal (grade 5) AE rates, and treatment-related grade 3/4 AEs rates were calculated as the number of each of these outcomes in the sub-study/arm divided by the total number of patients evaluated for response or toxicity in that sub-study/arm. Standard errors and confidence intervals (CIs) for a single proportion were derived. Pooled rates were estimated using meta-analysis for proportion. Modeling with random effects and the restricted maximum likelihood (REML) estimator were used to account for between-study heterogeneity. *I*^2^ statistics were calculated to provide a measure of the proportion of overall variation attributable to between-study heterogeneity. Meta-regression was used to explore potential sources of heterogeneity in rates related to (1) categories of therapy type in experimental sub-trials/arms and (2) types of sub-trials/arms (experimental vs non-match/control/placebo), as well as (3) study definition and (4) number of drugs tested. The results are presented as rates with 95% CI in each category and *p* value from the *Q* test for heterogeneity in meta-regression. The average number of treatment-related grade 3 and 4 AEs per person with a 95% confidence interval was estimated using a Poisson regression model. Unweighted median with 95% CI was calculated for PFS and OS by bootstrap methods [42] using the “boot” package in the R software.

Meta-analysis was conducted using the metafor package (R version 3.2.3); *p* < 0.05 was considered statistically significant. All tests were 2-sided.

## Results

**Fig. 2:**
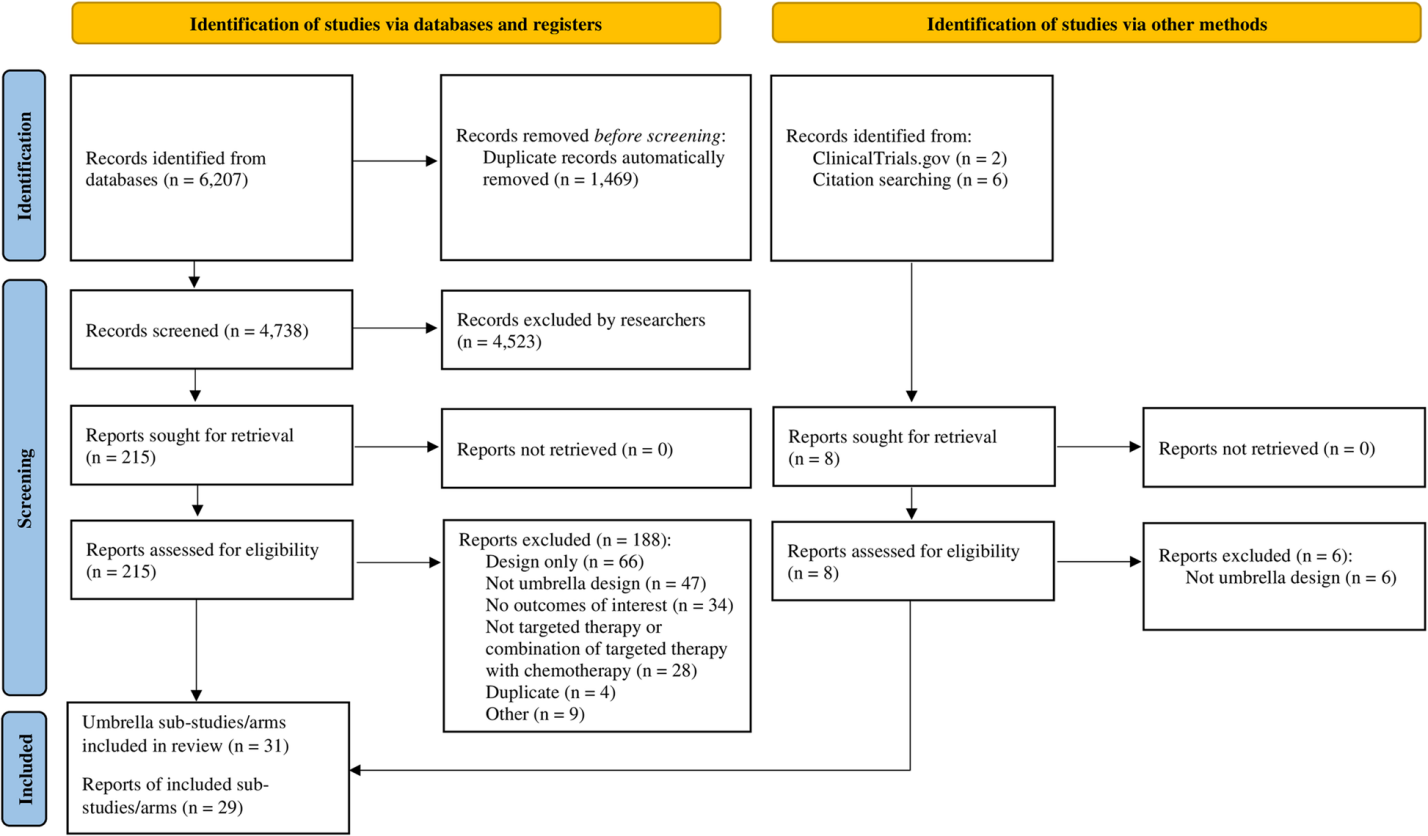
PRISMA 2020 flow diagram. The number of identified, screened, and included records with the exclusion reasons for potentially eligible reports

We retrieved 6207 references from searching databases. After duplicate removal, we screened 4738 records, from which we reviewed 215 full-text documents. In the next step, we searched references of the initially included studies and ClinicalTrials.gov entries and found 2 extra articles. Finally, we included 29 records of 31 sub-trials or arms of 9 umbrella trials. Figure 2 summarizes the search results and reasons for exclusion. A full list of included studies with therapy type, malignancy names, and agents tested is presented in the online appendix (Additional file 1: Table S3) [43–71].

### Trial and patient characteristics

We included 31 sub-trials or arms of nine umbrella trials (*N* = 1637). Six out of 9 umbrella trials can be also classified as platform umbrella trials (Additional file 1: Table S3). The majority (19; 61.2%) of included studies were sub-studies with a separate registration number, ten (32.3%) were umbrella trial arms without a separate registration number, and two (6.5%) were arms of the included sub-studies (Table 2). The majority (27; 87.1%) were biomarker-based experimental sub-studies/arms, two (6.5%) were non-biomarker specific, and one (3.2%) was either a placebo or a control group. Twenty-one sub-studies/arms (67.8%) tested targeted therapies, eight (25.8%) tested a combination of targeted therapy with chemotherapy, one (3.2%) tested standard-of-care chemotherapy, and one (3.2%) was a placebo arm. The majority of included sub-trials/arms (25; 80.6%) were phase II studies and tested one investigational drug (22; 71.0%). Six sub-trials/arms (19.4%) were funded by private sponsors, four (12.9%) by public institutions, and 21 (67.7%) by both private and public sponsors. Thirteen sub-trials/arms (42.0%) were conducted in North America, 12 (38.7%) in Asia, five (16.1%) in Europe, and one (3.2%) in Australia.

**Table 2:** Characteristics of the included studies

Characteristics	Category or number	Total (***n*** = 31), ***n (%)***
**Umbrella trial acronym and number of included sub-trials/arms**	Lung-MAP	5 (16.0^a^)
	BATTLE	4 (12.9)
	BATTLE-2	4 (12.9)
	Cluster trial	4 (12.9)
	SUKSES	4 (12.9)
	VIKTORY	4 (12.9)
	FOCUS4	2 (6.5)
	PICCOLO	2 (6.5)
	UmbHER1	2 (6.5)
**Study classification**	Sub-study	19 (61.2^a^)
	Umbrella trial arm	10 (32.3)
	Sub-study arm	2 (6.5)
**Sub-trial/arm type**	Experimental	27 (87.1)
	Experimental—non-match	2 (6.5)
	Control group	1 (3.2)
	Placebo	1 (3.2)
**Therapy type**	Targeted therapy	21 (67.8^a^)
	Targeted therapy with chemotherapy	8 (25.8)
	Chemotherapy	1 (3.2)
	Placebo	1 (3.2)
**Publication year**	2019	14 (45.1^a^)
	2018	7 (22.6)
	2016	4 (12.9)
	2013	4 (12.9)
	2011	2 (6.5)
**Location**	North America	13 (42.0^a^)
	Asia	12 (38.7)
	Europe	5 (16.1)
	Australia	1 (3.2)
**Funding**	Mixed	21 (67.7)
	Private	6 (19.4)
	Public	4 (12.9)
**Sub-trial/arm status**	Completed	20 (64.5)
	Closed at interim analysis	7 (22.5^a^)
	Terminated	2 (6.5)
	Unknown	2 (6.5)
**Study definition**	Phase II	25 (80.6)
	Phase III	4 (12.9)
	Phase II/III	2 (6.5)
**Total number of investigational drugs**	1 drug	22 (71.0)
	≥ 2 drugs	9 (29.0)
**Further studies recommended**	No	15 (48.3^a^)
	Not reported	8 (25.8)
	Yes	6 (19.4)
	Not applicable	2 (6.5)
**Enrolled patients,** ***n*** **(*%*)**	1637 (100)	
**Patients evaluable for toxicity,** ***n*** **(%)**^**b**^	1379 (84.2)	
**Patients evaluable for response,** ***n*** **(%)**^**c**^	1328 (81.1)	
**Male patients,** ***n*** **(%)**^**d**^	512 (31.3)	
**Median age at enrollment,** ***n*** **(%) of sub-studies/arms**	Not reported	13 (41.9)
	< 65	13 (41.9)
	≥ 65	5 (16.2^a^)
**Tumor type,** ***n*** **(%) of sub-studies/arms**	Solid tumors	31 (100)
	Hematological malignancies	0 (0)
**Stage of disease**	Stage IIIB or IV or advanced or metastatic or relapsed	30 (96.8)
	Early and locally advanced	1 (3.2)
**Performance status scale used,** ***n*** **(%) of sub-studies/arms**	ECOG/WHO/Zubrod 0–1	9 (29.0)
	ECOG/WHO/Zubrod 0–2	22 (71.0)

*ECOG* Eastern Cooperative Oncology Group, *WHO* World Health Organization

^a^ The percentage was calculated by subtracting the remaining % values from 100%

^b^ The number of patients evaluable for toxicity was not reported in 8 sub-trials/arms

^c^ The number of patients evaluable for response was not reported in 1 sub-trial

^d^ Sex was not reported in 13 sub-trials/arms

In 13 sub-studies/arms, the median age of participants was below 65 years; in 5 sub-studies/arms, the median age was 65 years or higher; and in the remaining 13 sub-studies/arms, the median age was not reported (Table 2). All sub-trials/arms involved only patients with solid tumors.

### Benefit in experimental sub-trials/arms

Twenty-two of 27 experimental sub-trials/arms reported response data. We identified 185 objective responses (including 142 partial and 5 complete reported separately and 38 reported as objective responses) among 879 participants evaluated for response. One targeted therapy experimental arm was excluded from the meta-analysis because only 1 patient was evaluated for response in that arm.

**Fig. 3:**
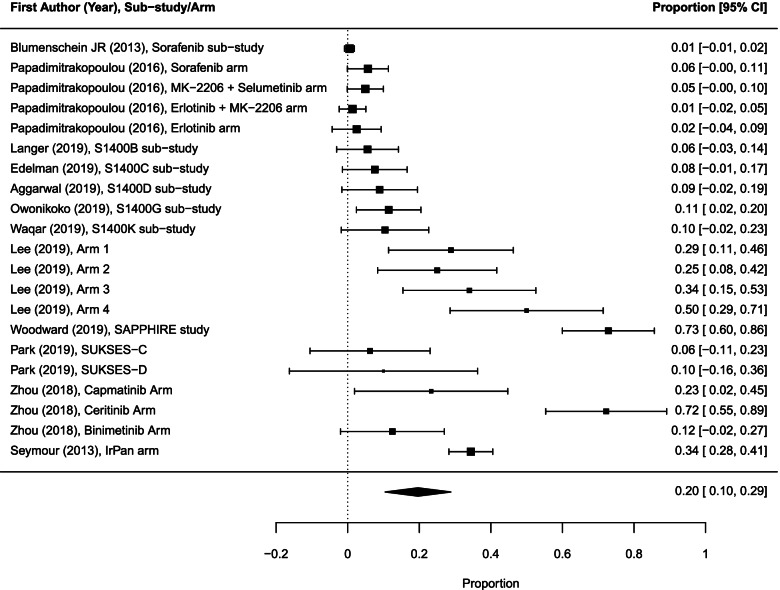
Forest plot of proportions of objective response rates in experimental sub-trials/arms included in the meta-analysis (random effects). The analysis included data from 21 experimental sub-trials/arms with a total of 185 objective responses among 878 participants evaluated for response. CI, confidence interval

The pooled ORR across 21 sub-trials/arms (878 patients) was 19.7% (95% CI 10.5–28.8; *I*^2^ = 97.3%; Fig. 3). The ORR for targeted therapies was significantly lower than the ORR for combination of targeted therapy drugs with chemotherapy: 13.3% (95% CI 4.6–21.9) vs 39.0% (95% CI 21.3–56.8), *p* = 0.005.

The median PFS ranging from 1.2 to 17.0 months was reported in 16 experimental sub-trials/arms [72]. The pooled median PFS was 2.5 months (95% CI 2.0–7.8).

The median OS ranging from 4.7 to 10.4 months was reported in 12 experimental sub-trials/arms. The pooled median OS was 6.8 months (95% CI 6.0–8.0). We did not compare pooled PFS and OS between targeted therapy and a combination of targeted therapy with chemotherapy because only one sub-study/arm testing combination of the therapies reported these outcomes.

### Risk in experimental sub-trials/arms

**Fig. 4:**
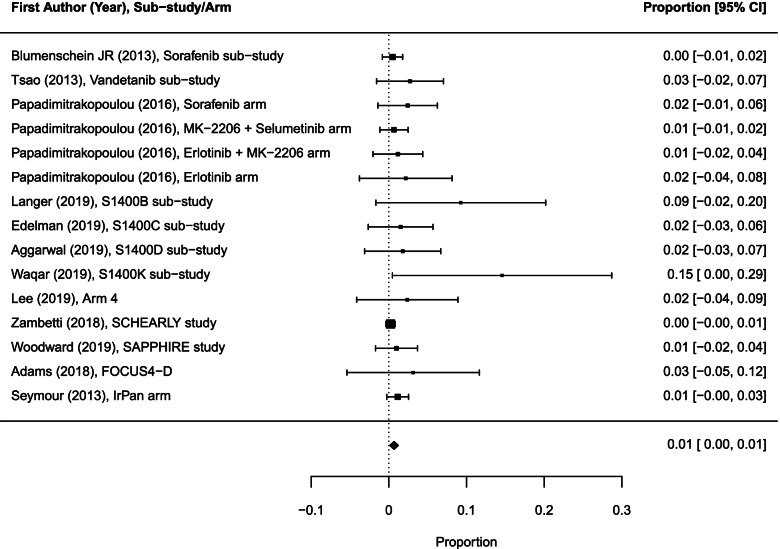
Forest plot of proportions of treatment-related grade 5 AEs in experimental sub-trials/arms included in the meta-analysis (random effects). The analysis included data from 15 experimental sub-trials/arms with a total of 9 drug-related deaths among 999 participants evaluated for toxicity. AEs, adverse events; CI, confidence interval

We analyzed 9 drug-related grade 5 AEs among 999 participants evaluated for toxicity in 15 experimental sub-trials/arms (including 9 drug-related deaths in 5 experimental sub-trials/arms and in the remaining 10 sub-trials/arms the drug-related deaths were reported to be 0). The pooled drug-related death rate across these sub-trials/arms was 0.7% (95% CI 0.1–1.2; *I*^2^ = 4.5%; Fig. 4).

The pooled drug-related grade 5 AE rate for 12 experimental targeted therapy sub-trials/arms (among 502 participants evaluated for toxicity) was 1.1% (95% CI 0.2–2.0) and for 3 experimental targeted therapy combined with chemotherapy sub-trials/arms (497 participants) was 0.5% (95% CI 0.1–1.2). Due to the small number of events, statistical comparisons between the different groups were not performed.

**Fig. 5:**
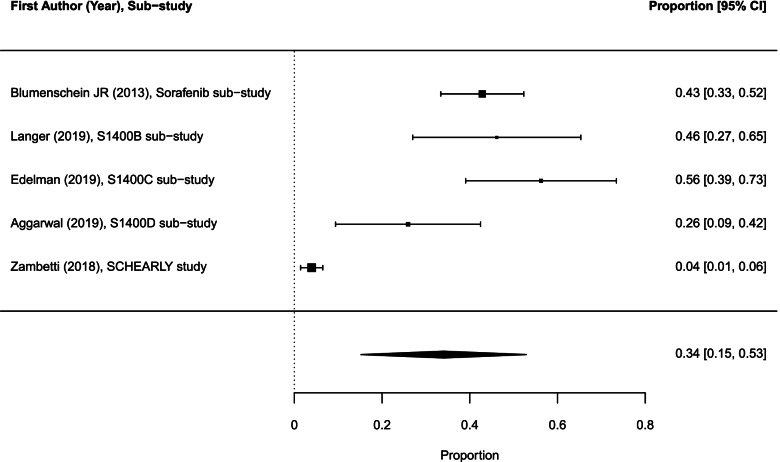
Forest plot of proportions of treatment-related grade 3/4 AEs in experimental sub-trials included in the meta-analysis (random effects). The analysis included data from 5 experimental sub-trials/arms with a total of 91 patients that experienced drug-related grade 3/4 AEs. AEs, adverse events; CI, confidence interval

Ninety-one patients (34.0%; 95% CI 15.2–52.9) experienced treatment-related grade 3/4 AEs in 5 sub-trials (Fig. 5). The treatment-related grade 3/4 rate in four of these sub-trials/arms testing targeted therapy drugs was 42.6% (95% CI 31.2–53.9).

Three hundred eleven drug-related grade 3/4 AEs were reported in 11 experimental sub-trials/arms (10 targeted therapy, one targeted therapy with chemotherapy) among 695 toxicity evaluable patients, with an average drug-related grade 3/4 AE rate per person of 0.45 (95% CI 0.40–0.50).

### Overall benefit and risk

**Fig. 6:**
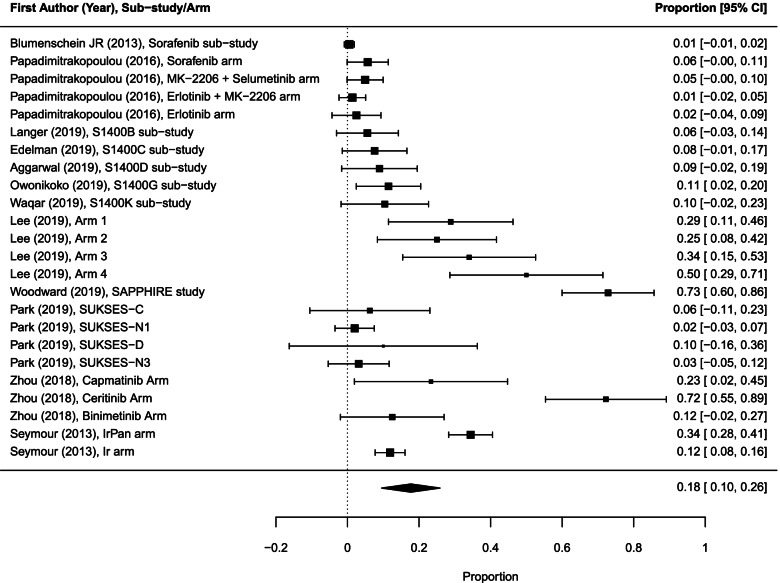
Forest plot of proportions of objective response rates in sub-trials/arms included in the meta-analysis (random effects). The analysis included data from 24 sub-trials/arms with a total of 212 objective responses among 1147 participants evaluated for response. CI, confidence interval

Twenty-five of 31 sub-trials/arms reported 212 objective responses (including 169 partial and 5 complete reported separately and 38 reported as objective responses) among 1148 participants evaluated for response. One arm was excluded from the meta-analysis because only 1 patient was evaluated for response in that arm. The pooled overall ORR across 24 sub-trials/arms (1147 patients) was 17.7% (95% CI 9.5–25.9; *I*^2^ = 97.3%; Fig. 6). We did not find a significant difference in ORR between experimental sub-trials/arms versus non-matched therapies: 19.7% (95% CI 10.5–28.8) vs 7.1% (95% CI 0.0–13.5); *p* = 0.25.

The median PFS ranging from 1.2 to 17.0 months was reported in 19 sub-trials/arms. The pooled median PFS was 2.4 months (95% CI 1.9–2.9). For 3 sub-trials/arms with non-matched therapies the pooled median PFS was 2.0 months (95% CI 1.2–3.5).

The median OS ranging from 4.7 to 10.5 months was reported in 13 sub-trials/arms. The pooled median OS was 7.1 months (95% CI 6.1–8.4). We did not compare the pooled OS rates between sub-trial/arm types because there was only one arm reporting this outcome in the non-matched group.

**Fig. 7:**
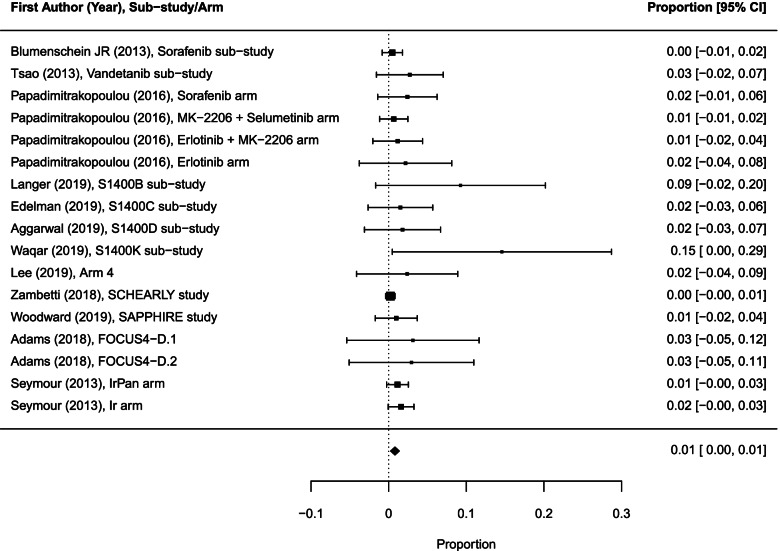
Forest plot of proportions of treatment-related grade 5 AEs in 17 sub-trials/arms included in the meta-analysis (random effects). The analysis included data from 17 sub-trials/arms with a total of 12 drug-related deaths among 1233 participants evaluated for toxicity. AEs, adverse events; CI, confidence interval

We identified 12 drug-related grade 5 AEs among 1233 patients evaluable for toxicity in 17 sub-trials/arms. The overall pooled drug-related death rate across these sub-trials/arms was 0.8% (95% CI 0.3–1.4; *I*^2^ = 7.32%; Fig. 7). We did not compare pooled drug-related death rates between sub-trial/arm types because there were two sub-trials/arms reporting this outcome in the non-match sub-category.

Treatment-related grade 3/4 AEs were not reported in non-matched sub-trials/arms. The overall treatment-related grade 3/4 AE rate and the average drug-related grade 3/4 AE rate per person remain the same as for the experimental arms described previously.

### Sub-group analysis of benefit and risk

We observed significant differences in ORR between Phase II and Phase III trials: 14.0% (95% CI 6.9-21.2) vs 36.4% (95% CI 3.4-69.3); *p* = 0.03 (Additional file 1: Table S4). Other benefit and risk measures in sub-categories are shown in the online appendix (Additional file 1: Table S4).

### Risk of bias assessment

The risk of bias of the included sub-studies/arms is available in the online appendix (Figs. S1-S4). There were two randomized sub-studies with “low” or “some concerns” risk of bias among all domains. There were 27 nonrandomized sub-trials/arms, and 16 of them (59%) were assessed as having the overall risk of bias as “serious” or “critical.” High levels of bias were mainly due to bias in the selection of the reported result.

## Discussion

To our knowledge, we report the first systematic review and meta-analysis providing risk and benefit estimates for cancer umbrella trials testing targeted therapies or a combination of targeted therapies with chemotherapy. These analyses include four aspects: (1) the utility of a new strategy of clinical trials (umbrella designs) in oncology, (2) the utility of precision oncology, (3) the utility of pooling populations across arms and across chemotherapies, and (4) the likelihood that a drug works in more than one specific population.

Maximizing benefit and minimizing harm for participants of clinical research are one of the crucial ethical requirements. Umbrella trial designs are believed not only to be more flexible than traditional designs by allowing simultaneous evaluation of multiple treatment options but also to have a better risk/benefit ratio for trial participants [10]. However, our findings do not support the expectations of an increased benefit/risk ratio for participants of cancer umbrella trials.

Normally, the risk-benefit ratio in clinical trials is a function of the drug. However, clinical trial design may also affect this equation. Some elements of the umbrella design are expected to provide a more favorable risk/benefit ratio for participants than the classical trial designs. For example, the “precision medicine” approach of assigning patients to treatment arms based on the genetic characteristics of their tumor may lead to an expectation of higher responses and lower adverse event rates. Moreover, most of the umbrella trials in our study used the platform design and an adaptive design. This means that more patients may be enrolled in an arm with more promising health benefits, during the course of the study. Our results cast doubts on such an assumption. For example, the majority of the sub-trials/arms in our sample were phase II trials with pooled ORR of 14.0%. This result is similar to the pooled ORR in the previous meta-analysis of phase II single-agent studies (12.7%) [27] but lower than the overall ORR in eight cancer basket trials published until 31 March 2018 (25%) [73].

Our findings suggest that in all umbrella clinical trials in oncology published before 7 October 2019, the chances of responding from targeted therapies were lower than from the combination of targeted therapies with chemotherapy. This observation is consistent with the previous findings showing the increased overall response rates in trials testing a combination of targeted therapies with cytotoxic drugs [32]. Unfortunately, a comparison of the overall benefits and risks in our meta-analysis to other studies is limited as the included sub-trials/arms were very heterogeneous, e.g., they were of different phases, and included heterogeneous populations and various types of cancers.

The majority of the included sub-trials/arms reported surrogate outcomes: ORR (25; 80.6%) or PFS (19; 61.3%). Thirteen sub-trials/arms (41.9%) reported OS. A variety of endpoints makes a comparison of outcomes in meta-research more demanding. Importantly, although sometimes accepted by regulatory agencies for approval, ORR and PFS are surrogate markers that have shown poor correlation with OS and quality of life in most tumor types. Furthermore, early phases of clinical trials also poorly predict phase III success. Therefore, surrogate measures should be considered only hypothesis-generating and not a marker of true clinical benefit [74–78].

We did not find significant differences in objective response rates between therapies matched to the specific cancer biomarkers versus non-matched therapies or controls. This finding may suggest that the approaches to maximize the direct benefit in umbrella trials, e.g., genome-driven stratification and assignment to the most promising arm, may not be sufficient to deliver the appropriate therapy matching the heterogeneous and mutable tumor [8]. This also raises the question if biomarkers used to define the target in precision oncology may be suboptimal [79]. However, other systematic reviews analyzing pediatric phase I [80] and phase II [27] cancer trials showed high objective response rates in trials with target-specific enrolment [80] or in trials adopting personalized treatment approach [27].

The drug-related death rate of 1.1% in phase II sub-trials/arms in our sample (Additional file 1: Table S4) is also similar to the drug-related grade 5 AEs rate in the previous meta-analysis of phase II single-agent studies that used personalized strategy (1.5%) [27]. This may indicate that phase II umbrella trials do not offer a lower risk for trial participants than classical phase II clinical trials.

Debates about whether precision medicine is an illusion or an objective reality continues in oncology [81]. Our findings do not support the expectation of increased participants’ benefit in cancer umbrella trials. Patients should be clearly informed that the majority of participants (82.3%) of the first launched umbrella trials testing targeted therapy agents or combination of targeted therapy drugs with chemotherapy did not respond to a given therapy.

The objective of our study was to analyze the risk/benefit ratio for umbrella clinical trials testing targeted drugs or a combination of targeted therapy with chemotherapy. Analyses of that type are crucial sources of information for participants, researchers, ethics committee members, and other stakeholders and decision-makers. When performing our analysis, we found that the complexity of the umbrella design and the low quality of reporting makes a comparison of the results from trials and sub-trials very difficult. Many umbrella arms and sub-trials of the umbrella trials included in our study were closed without any explanation and without a report of the results. Our findings may be used to improve umbrella trials design reporting.

## Limitations

Our study should be interpreted in light of the following limitations.

First, we included all umbrella trials or sub-trials being a part of one umbrella trial in which one cancer type was divided into sub-types to test the different drugs. Because umbrella trials are very heterogeneous and have a hierarchical structure and every arm may be of different phase, we did not compare the whole umbrella trials but compared the sub-trials and arms. We created this novel methodology to analyze the outcomes of a complex umbrella trial design but our methods may be further modified and improved.

Second, we observed inconsistency in reporting the outcomes and selective reporting of the results in the majority of the included umbrella trials. For example, in the VIKTORY trial [71], outcomes were not provided for six out of 10 arms. Low quality of reporting of umbrella trials is a serious issue not only because of the difficulties in performing meta-research but also for other ethical reasons, including patients’ safety and decision-making process in designing new trials.

Third, ORR and PFS reported in most umbrella trials are considered a surrogate benefit and are not markers of direct clinical benefit [76, 82]. We analyze them because they are the best available surrogates of clinical benefit.

Fourth, our systematic review is restricted to umbrella trials which results were reported between 2006 and 2019 and does not include trials developed based on the findings of those initial trials. As relatively novel designs, umbrella trials are expected to improve methodologically over time and may produce results leading to different conclusions than those presented here.

Fifth, the limited number of eligible sub-trials reporting the outcomes of interest did not allow for all possible comparisons, for example, pooled PFS and OS.

Sixth, the majority of the analyzed studies reported summary results. Thus, we could not test whether risk and benefit in umbrella trials depend on the line of treatment or cancer histology.

## Conclusions

This is the first systematic review with meta-analysis assessing the risk and benefits of umbrella clinical trials. We found that the overall objective response rate in umbrella trials testing targeted drugs or a combination of targeted therapy with chemotherapy was 17.7%, and the overall drug-related death rate was 0.8%. Patients enrolling in umbrella trials should be clearly informed about the risk and benefit predictions for these trials. Our findings do not support the expectation of increased patients’ benefits in cancer umbrella trials. Further studies should investigate whether umbrella trial design and precision oncology approach improve patient outcomes. Our study identified serious problems with reporting and transparency of umbrella design which may undermine a promise of more efficient and patient-centered trials.

### Registration and protocol

The study protocol was prospectively registered in PROSPERO (CRD42020171494) [36].

## Supplementary Information

**Additional file 1: Table S1**. Search strategy. **Table S2**. Glossary of key manuscript terms. **Table S3**. List and characteristics of included studies. **Table S4**. Objective response rates and drug-related fatal toxicity rates assessed in subgroups. **Fig. S1**. Summary risk of bias graph of randomized sub-studies. **Fig. S2**. Review authors’ judgements about risk of bias of randomized sub-studies. **Fig. S3**. Summary risk of bias graph of all non-randomized sub-studies/arms. **Fig. S4**. Review authors’ judgements about risk of bias for non-randomized sub-studies/arms.

## Data Availability

Data are available in the Open Science Framework online public database (https://osf.io/uc5ds/). Additional data are available upon request.

## Abbreviations

AEs: Adverse events
CI: Confidence interval
ECOG: Eastern Cooperative Oncology Group
OS: Overall survival
ORR: Objective response rate
OSF: Open Science Framework
PFS: Progression-free survival
REML: Restricted maximum likelihood
WHO: World Health Organization
