# Supplementary material for: Risk and benefit for umbrella trials in oncology: a systematic review and meta-analysis
Source: BMC Med. 2022 Jul 8;20:219. doi: 10.1186/s12916-022-02420-2 (PMC9264503; doi:10.1186/s12916-022-02420-2)
Supplement: Supplementary file 1 — Additional file 1: Table S1. Search strategy. Table S2. Glossary of key manuscript terms. Table S3. List and characteristics of included studies. Table S4. Objective response rates and drug-related fatal toxicity rates assessed in subgroups. Fig. S1. Summary risk of bias graph of randomized sub-studies. Fig. S2. Review authors’ judgements about risk of bias of randomized sub-studies. Fig. S3. Summary risk of bias graph of all non-randomized sub-studies/arms. Fig. S4. Review authors’ judgements about risk of bias for non-randomized sub-studies/arms. [file 12916_2022_2420_MOESM1_ESM.docx]

**Supplementary Information**

[Table S1. Search strategy. 2](#_Toc104542259)

[Table S2. Glossary of key manuscript terms. 3](#_Toc104542260)

[Table S3. List and characteristics of included studies. 4](#_Toc104542261)

[Table S4. Objective response rates and drug-related fatal toxicity rates assessed in subgroups. 6](#_Toc104542262)

[Figure S1. Summary risk of bias graph of randomized sub-studies. 7](#_Toc104542263)

[Figure S2. Review authors’ judgements about risk of bias of randomized sub-studies. 7](#_Toc104542264)

[Figure S3. Summary risk of bias graph of all non-randomized sub-studies/arms. 7](#_Toc104542265)

[Figure S4. Review authors’ judgements about risk of bias for non-randomized sub-studies/arms. 8](#_Toc104542266)

# **Table S1. Search strategy.**

| **PubMed (2035 items)** |
| --- |
| Search ((((((((((((((((((((((((((((((((((((((((((((((((((((((((cancer) OR cancers) OR carcinoma) OR cancerous) OR carcinom*) OR neoplasm) OR neoplas*) OR oncology) OR oncolog*) OR tumor) OR tumors) OR solid tumor) OR solid tumors) OR malignancy) OR malignan*) OR leukemia) OR leukemias) OR leucocythemia) OR acute myeloid leukemia) OR acute myeloid leukaemia) OR acute lymphoblastic leukemia) OR acute myelogenous leukemia) OR hematologic malignancy) OR hematologic neoplasms) OR chronic myelogenous leukemia) OR juvenile myelomonocytic leukemia) OR juvenile myelomonocytic leukaemia) OR myelodysplastic syndrome) OR myelodysplastic syndromes) OR transient myeloproliferative disorder) OR myeloid malignancies) OR myeloid malignancy) OR lymphoma) OR hodgkin disease) OR hodgkins disease) OR sarcoma) OR osteosarcoma) OR ewing sarcoma) OR ewing tumor) OR rhabdomyosarcoma) OR wilms tumor) OR nephroblastoma) OR retinoblastoma) OR medulloblastoma) OR teratoma) OR germinoma) OR germ cell tumor) OR dysgerminoma) OR seminoma) OR gonadoblastoma) OR *glioma) OR glioblastoma) OR astrocytoma) OR *carcinoma) OR *blastoma)) AND ((((((((((((((((((((((((((((((Umbrella) OR Master protocol) OR Master protocols) OR “Platform trial”) OR “Platform trials”) OR “Platform study”) OR “Platform studies”) OR Subprotocol) OR Sub-protocol) OR Sub-protocols) OR Subprotocols) OR Substudy) OR Substudies) OR Sub-study) OR Sub-studies) OR Sub-trial) OR Subtrial) OR Sub-trials) OR Subtrials) OR Substratified) OR Sub-stratified) OR Molecularly stratified) OR Biomarker-stratified) OR Biomarker-integrated) OR Biomarker-driven) OR Biomarker-directed) OR Biomarker-specific) OR Marker-driven) OR Genome-driven) OR Genomics-driven) Filters: Publication date from 2006/01/01 to 2019/10/07 |
| **Embase (4172 items)** |
| ((cancer OR cancers OR carcinoma OR cancerous OR carcinom* OR neoplasm OR neoplas* OR oncology OR oncolog* OR tumor OR tumour OR tumors OR tumours OR 'solid tumor' OR 'solid tumors' OR 'solid tumour' OR 'solid tumours' OR malignancy OR malignan* OR leukemia OR leukaemia OR leukemias OR leukaemias OR leucocythaemia OR leucocythemia OR (acute AND myeloid AND leukemia) OR (acute AND myeloid AND leukaemia) OR (acute AND lymphoblastic AND leukemia) OR (acute AND lymphoblastic AND leukaemia) OR (acute AND myelogenous AND leukemia) OR (acute AND myelogenous AND leukaemia) OR (hematologic AND malignancy) OR (haematologic AND malignancy) OR (hematologic AND neoplasms) OR (haematologic AND neoplasms) OR (chronic AND myelogenous AND leukemia) OR (chronic AND myelogenous AND leukaemia) OR (juvenile AND myelomonocytic AND leukemia) OR (juvenile AND myelomonocytic AND leukaemia) OR (myelodysplastic AND syndrome) OR (myelodysplastic AND syndromes) OR (transient AND myeloproliferative AND disorder) OR (myeloid AND malignancies) OR (myeloid AND malignancy) OR lymphoma OR (hodgkin AND disease) OR (hodgkins AND disease) OR sarcoma OR osteosarcoma OR (ewing AND sarcoma) OR (ewing AND tumor) OR (ewing AND tumour) OR rhabdomyosarcoma OR (wilms AND tumour) OR (wilms AND tumor) OR nephroblastoma OR retinoblastoma OR medulloblastoma OR teratoma OR germinoma OR (germ AND cell AND tumor) OR (germ AND cell AND tumour) OR dysgerminoma OR seminoma OR gonadoblastoma OR $glioma OR glioblastoma OR astrocytoma OR $carcinoma OR $blastoma) AND (umbrella OR 'master protocol' OR 'master protocols' OR 'platform trial' OR 'platform trials' OR 'platform study' OR 'platform studies' OR subprotocol OR 'sub protocol' OR 'sub protocols' OR subprotocols OR substudy OR substudies OR 'sub study' OR 'sub studies' OR 'sub trial' OR subtrial OR 'sub trials' OR subtrials OR substratified OR 'sub stratified' OR 'molecularly stratified' OR 'biomarker stratified' OR 'biomarker driven' OR 'biomarker integrated' OR 'biomarker directed' OR 'biomarker specific' OR 'marker driven' OR 'genome driven' OR 'genomics driven')) AND (2006:py OR 2007:py OR 2008:py OR 2009:py OR 2010:py OR 2011:py OR 2012:py OR 2013:py OR 2014:py OR 2015:py OR 2016:py OR 2017:py OR 2018:py OR 2019:py) |
| **Search date: 7^th^ October 2019** |

# **Table S2. Glossary of key manuscript terms.**

| **Adverse event (AE)** | any unfavorable and unintended sign (including an abnormal laboratory finding), symptom, or disease temporally associated with the use of a medical treatment or procedure that may or may not be considered related to the medical treatment or procedure. |
| --- | --- |
| **Molecular profiling** | method of testing genetic characteristics as well as any unique biomarkers of a cancerous tumor. The results are used to identify and create targeted therapies that work most effectively for specific cancer tumor profiles. |
| **Non-match arm/sub-study** | arm or sub-study recruiting patients that did not match any of the prespecified biomarkers tested in umbrella trial. |
| **Objective response rate (ORR)** | proportion of participants with partial and/or complete response (reported separately or as an objective response rate). |
| **Precision oncology** | strategy aiming to divide cancer patients into groups that will most likely respond to a given therapy. Treatment is tailored to the molecular makeup of a tumor rather than the site or stage of disease. |
| **Sub-study (sub-trial)** | rate trial within the umbrella protocol with a unique registration number. |
| **Umbrella platform trial** | umbrella trial that permits considerable flexibility, for example, to add new arms when novel targets and drugs are identified or to discontinue arms with ineffective treatments. It usually utilizes Bayesian response-adaptive randomization. |
| **Umbrella trial** | type of master protocol with many different treatment arms within one trial. People are assigned to a particular treatment arm of the trial based on their type of cancer and the specific molecular makeup of their cancer. |

# **Table S3. List and characteristics of included studies.**

| **No.** | **Name of the umbrella trial** | **Type of umbrella trial** | **Tumor type name** | **Phase*** | **Name of included sub-trial/arm** | **Sub-trial/arm type** | **Number of enrolled patients** | **Drug name/s** | **Therapy type** | **Reference/s** |
| --- | --- | --- | --- | --- | --- | --- | --- | --- | --- | --- |
| 1 | **BATTLE** | Platform umbrella trial | Non–small-cell lung cancer (NSCLC) | II | Erlotinib + bexarotene sub-study | Experimental | 37 | Bexarotene, erlotinib | Targeted therapy + chemotherapy | [43,44] |
| 2 |  |  |  |  | Erlotinib sub-study | Experimental | 59 | Erlotinib | Targeted therapy |  |
| 3 |  |  |  |  | Sorafenib sub-study | Experimental | 105 | Sorafenib | Targeted therapy | [43-46] |
| 4 |  |  |  |  | Vandetanib sub-study | Experimental | 54 | Vandetanib | Targeted therapy | [43,44,47] |
| 5 | **BATTLE-2** | Platform umbrella trial | Non–small-cell lung cancer (NSCLC) | II | Erlotinib arm | Experimental | 22 | Erlotinib | Targeted therapy | [48,49] |
| 6 |  |  |  |  | Erlotinib + MK-2206 arm | Experimental | 42 | Erlotinib, MK-2206 | Targeted therapy |  |
| 7 |  |  |  |  | MK-2206 + Selumetinib arm | Experimental | 75 | MK-2206, selumetinib | Targeted therapy |  |
| 8 |  |  |  |  | Sorafenib arm | Experimental | 61 | Sorafenib | Targeted therapy |  |
| 9 | **Cluster Trial** | Umbrella trial | Non-small-cell lung cancer (NSCLC) | II | Alpelisib Arm | Experimental | 2 | Alpelisib (BYL719) | Targeted therapy | [50,51] |
| 10 |  |  |  |  | Binimetinib Arm | Experimental | 22 | Binimetinib (MEK162) | Targeted therapy |  |
| 11 |  |  |  |  | Capmatinib Arm | Experimental | 16 | Capmatinib (INC280) | Targeted therapy |  |
| 12 |  |  |  |  | Ceritinib Arm | Experimental | 26 | Ceritinib (LDK378) | Targeted therapy |  |
| 13 | **FOCUS4** | Platform umbrella trial | Colorectal cancer | II/III | FOCUS4-D | Experimental | 16 | Sapitinib (AZD8931) | Targeted therapy | [52-54] |
| 14 |  |  |  |  | FOCUS4-D | Placebo | 16 | Placebo | Placebo |  |
| 15 | **Lung-MAP** | Platform umbrella trial | Squamous cell lung cancer (SCC) | II | S1400B sub-study | Experimental | 31 | GDC-0032 (Taselisib) | Targeted therapy | [55-57] |
| 16 |  |  |  |  | S1400C sub-study | Experimental | 37 | Palbociclib | Targeted therapy | [55,58,59] |
| 17 |  |  |  |  | S1400D sub-study | Experimental | 43 | AZD4547 | Targeted therapy | [55,60,61] |
| 18 |  |  |  |  | S1400G sub-study | Experimental | 51 | Talazoparib (BMN 673) | Targeted therapy | [62] |
| 19 |  |  |  |  | S1400K sub-study | Experimental | 28 | Telisotuzumab vedotin (ABBV-399) | Targeted therapy | [63] |
| 20 | **PICCOLO** | Umbrella trial | Colorectal cancer | III | IrPan arm | Experimental | 230 | Panitumumab + irinotecan | Targeted therapy + chemotherapy | [64] |
| 21 |  |  |  |  | Ir arm | Control group | 230 | Irinotecan | Chemotherapy |  |
| 22 | **SUKSES** | Platform umbrella trial | Small-cell lung cancer (SCLC) | II | SUKSES-C | Experimental | 7 | Adavosertib (AZD1775) | Targeted therapy | [65] |
| 23 |  |  |  |  | SUKSES-D | Experimental | 4 | Vistusertib (AZD2014) | Targeted therapy |  |
| 24 |  |  |  |  | SUKSES-N1 | Non-match | 24 | Adavosertib (AZD1775) | Targeted therapy |  |
| 25 |  |  |  |  | SUKSES-N3 | Non-match | 15 | Barasertib (AZD2811) | Targeted therapy |  |
| 26 | **UmbHER1** | Umbrella trial | Breast cancer | IIIB | SCHEARLY study | Experimental | 240 | Trastuzumab + conventional CT | Targeted therapy + chemotherapy | [66-68] |
| 27 |  |  |  |  | SAPPHIRE study | Experimental | 50 | Pertuzumab + trastuzumab + CT (nab-paclitaxel, docetaxel, or paclitaxel) | Targeted therapy + chemotherapy | [66,67,69] |
| 28 | **VIKTORY** | Platform umbrella trial | Gastric cancer | II | Arm 1 | Experimental | 25 | Selumetinib (AZD6244) + docetaxel | Targeted therapy + chemotherapy | [70,71] |
| 29 |  |  |  |  | Arm 2 | Experimental | 25 | Adavosertib (AZD1775) + paclitaxel | Targeted therapy + chemotherapy | [71] |
| 30 |  |  |  |  | Arm 3 | Experimental | 24 | Capivasertib (AZD5363) + paclitaxel | Targeted therapy + chemotherapy | [71] |
| 31 |  |  |  |  | Arm 4 | Experimental | 20 | Savolitinib (AZD6094 or volitinib) | Targeted therapy + chemotherapy | [71] |
| *In principle each of sub-trial within umbrella design could be of different phase. In our sample all included sub-trials within umbrella design were of the same phase. The phase presented in table does not represent all possible phases within included umbrella trial. For example, Arm 5 of VIKTORY umbrella trial was Phase I//II sub-trial and Arm 8 was a Phase I sub-trial.  CT – chemotherapy. | | | | | | | | | | |

# **Table S4. Objective response rates and drug-related fatal toxicity rates assessed in subgroups.**

| **Outcome** | **Measure** | **Subgroup** | | | | |
| --- | --- | --- | --- | --- | --- | --- |
|  |  | **Study definition** | | | **Total number of investigational drugs** | |
|  |  | **Phase 2** | **Phase 3** | **Phase 2/3** | **1 drug** | **≥2 drugs** |
| **Objective response** | **No. of studies** | 21 | 3 | ‒ | 17 | 7 |
|  | **Response rate, % (95% CI)** | 14.0 (6.9-21.2) | 36.4 (3.4-69.3) | NR | 13.0 (5.0-21.1) | 28.3 (10.4-46.2) |
|  | ***p*-Value** | 0.03* | | ‒ | 0.08* | |
| **Drug-related fatal (grade 5) AEs** | **No. of studies** | 11 | 4 | 2 | 12 | 5 |
|  | **Drug-related grade 5 AE rate, % (95% CI)** | 1.1 (0.2-1.9) | 0.7 (0.0-1.5) | 0† | 1.4 (0.5-2.4) | 0.5 (0.1-1.0) |
|  | ***p*-Value** | ‒ | | | 0.0987* | |
| * *p*-Value from Q test for heterogeneity comparing response rates and drug-related grade 5 AE rates between subgroups. † Two studies not meta-analyzed due to 0 drug-related deaths.  AE - adverse event; CI - confidence interval; NR - not reported. | | | | | | |

**Risk of bias of randomized and non-randomized sub-studies/arms**

All the plots were generated by robvis visualization tool available on: <https://www.riskofbias.info/welcome/robvis-visualization-tool>.


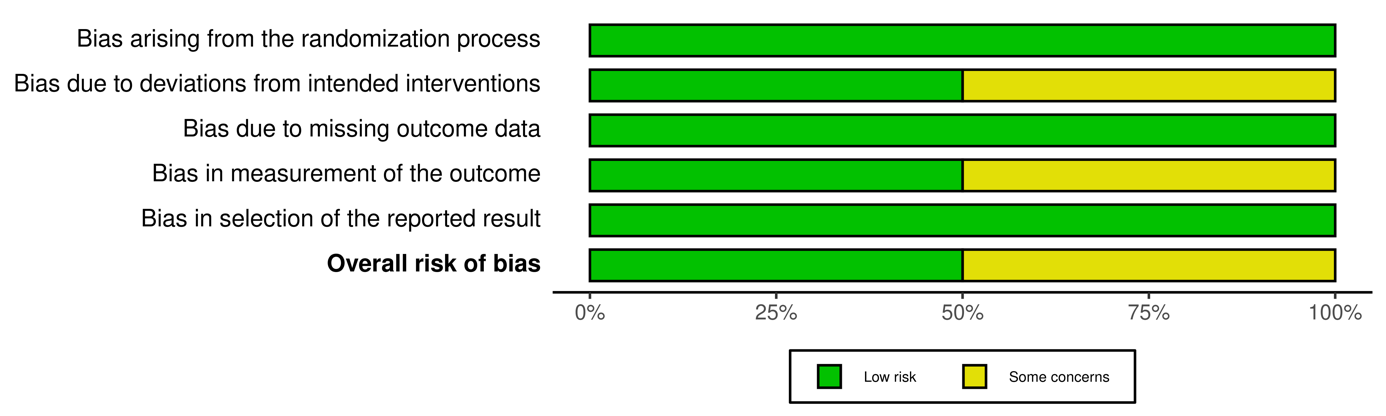


# **Figure S1. Summary risk of bias graph of randomized sub-studies.**

Risk of bias was assessed using RoB 2 tool - a revised Cochrane risk of bias tool for randomized trials [40].

**
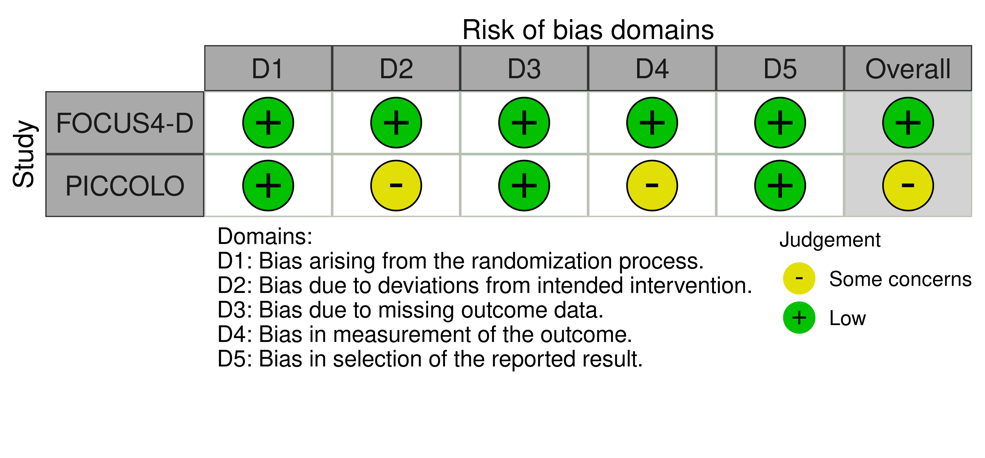
**

# **Figure S2. Review authors’ judgements about risk of bias of randomized sub-studies.**

Risk of bias was assessed using RoB 2 tool - a revised Cochrane risk of bias tool for randomized trials [40].


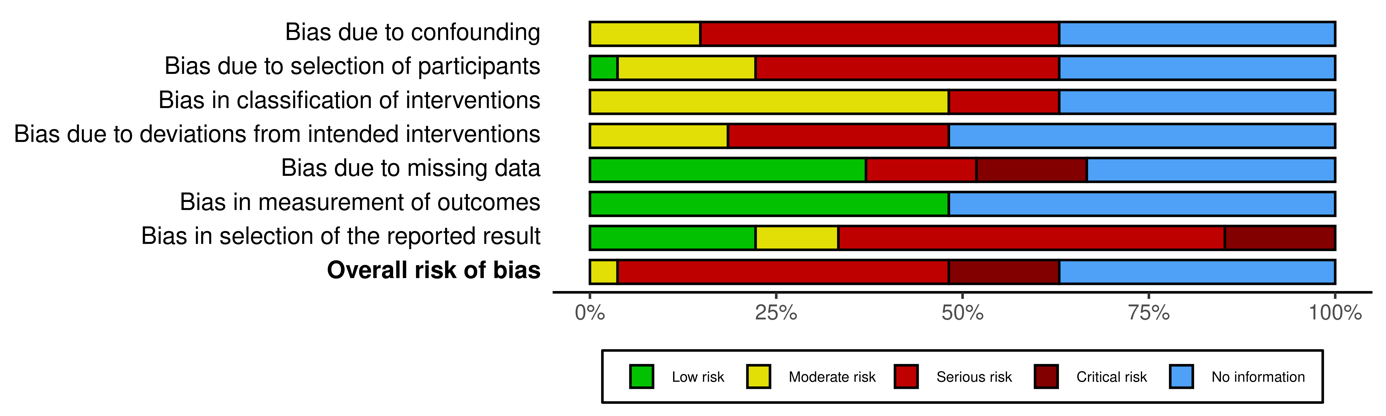


# **Figure S3. Summary risk of bias graph of all non-randomized sub-studies/arms.**

Risk of bias was assessed using ROBINS-I tool [41].


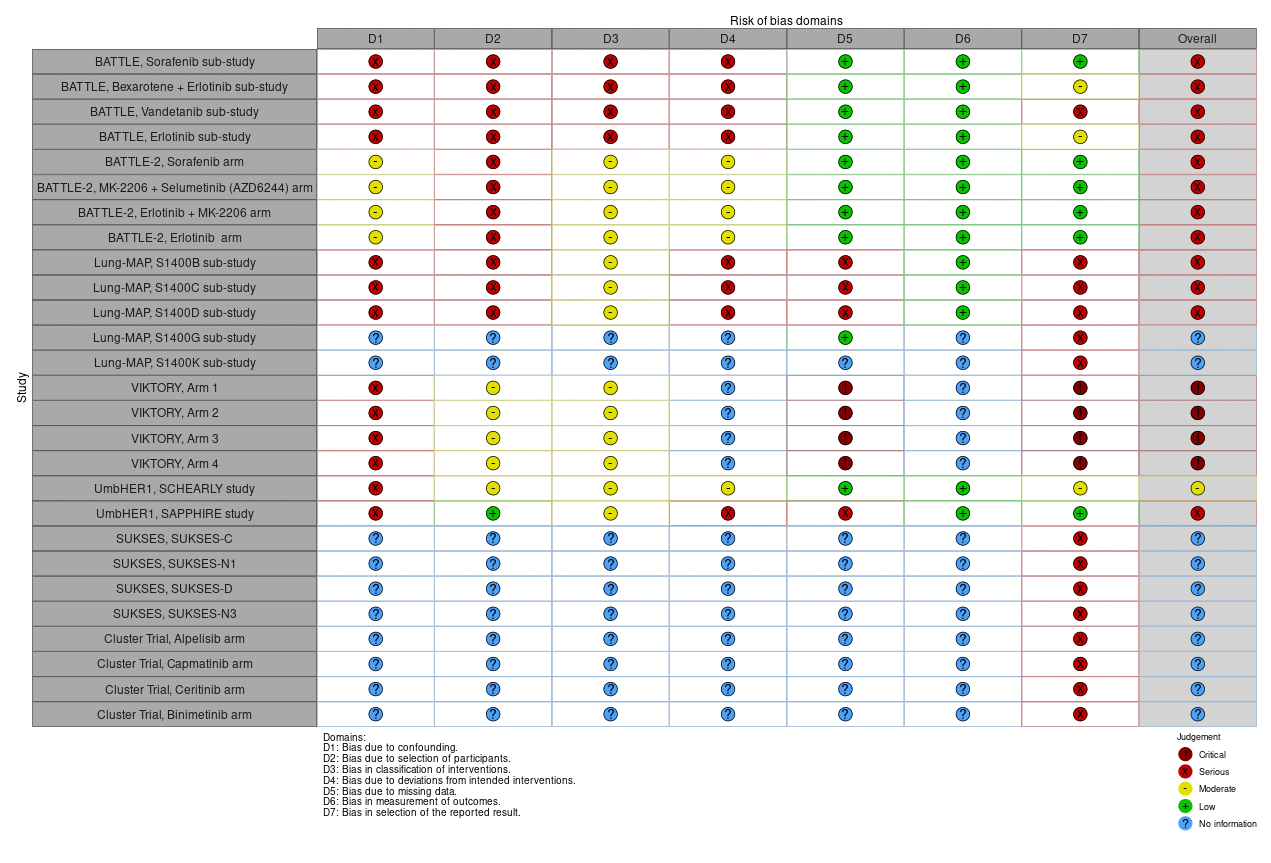


# **Figure S4. Review authors’ judgements about risk of bias for non-randomized sub-studies/arms.**

Risk of bias was assessed using ROBINS-I tool [41].
